# Hand, Foot, and Mouth Disease Risk Prediction in Southern China: Time Series Study Integrating Web-Based Search and Epidemiological Surveillance Data

**DOI:** 10.2196/75434

**Published:** 2025-10-09

**Authors:** Yixiong Chen, Xue Zhang, Sheng Zhang, Wenjie Han, Ziqi Wang, Jian Chen, Jinfeng Liu, Jingru Feng, Jiayi Shi, Haoyu Long, Zicheng Cao, Jie Zhang, Yuan Li, Xiangjun Du, Xindong Zhang, Meng Ren

**Affiliations:** 1 Bao’an Center for Disease Control and Prevention, 3 Haixiu Road, Bao'an District, Shenzhen, 518101, China, 86 0755-27757972; 2 School of Public Health (Shenzhen), Sun Yat-sen University, Guangzhou, China; 3 School of Public Health (Shenzhen), Shenzhen Campus of Sun Yat-sen University, Shenzhen, China; 4 Guangdong Provincial Center for Disease Control and Prevention, Guangzhou, China; 5 School of Public Health, Shantou University, Shantou, China; 6 Shenzhen Key Laboratory of Pathogenic Microbes & Biosafety, Shenzhen Campus of Sun Yat-sen University, Shenzhen, China; 7 Key Laboratory of Tropical Disease Control, Ministry of Education, Sun Yat-sen University, Guangzhou, China; 8 Shenzhen Field Epidemiology Training Project, Shenzhen, China

**Keywords:** HFMD, meteorological factors, Baidu Index, forecast, risk assessment, hand, foot, and mouth disease

## Abstract

**Background:**

Hand, foot, and mouth disease (HFMD) is a global health concern requiring a risk assessment framework based on systematic factors analysis for prevention and control.

**Objective:**

This study aims to construct a comprehensive HFMD risk assessment framework by integrating multisource data, including historical incidence information, environmental parameters, and web-based search behavior data, to improve predictive performance.

**Methods:**

We integrated multisource data (HFMD cases, meteorology, air pollution, Baidu Index, and public health measures) from Bao’an District of Shenzhen city in Southern China (2014‐2023). Correlation analysis was used to assess the associations between HFMD incidence and systematic factors. The impacts of environmental factors were analyzed using the Distributed Lag Nonlinear Model. Seasonal Autoregressive Integrated Moving Average model and advanced machine learning methods were used to predict HFMD 1-4 weeks ahead. Risk levels for the 1- to 4-week-ahead forecasts were determined by comparing the predicted weekly incidence against predefined thresholds.

**Results:**

From 2014 to 2023, Bao’an District reported a total of 118,826 cases of HFMD. Environmental and search behavior factors (excluding sulfur dioxide) were significantly associated with HFMD incidence in nonlinear patterns. For 1-week-ahead prediction, Seasonal Autoregressive Integrated Moving Average using case data alone performed best (*R*²=0.95, *r*=0.98, mean absolute error=53.34, and root-mean-square error=99.31). For 2- to 4-week-ahead forecasting, machine learning models incorporating web-based and environmental data showed superior performance (*R*²=0.83, 0.75, and 0.64; *r*=0.92, 0.87, and 0.80; mean absolute error=87.84, 112.41, and 132.47; and root-mean-square error=185.08, 229.13, and 276.81). The predicted HFMD risk levels matched the observed levels with accuracies of 96%, 87%, 88%, and 83%, respectively.

**Conclusions:**

The epidemic dynamics of HFMD are influenced by multiple factors in a nonlinear manner. Integrating multisource data, particularly web-based search behavior, significantly enhances the accuracy of short- and midterm forecasts and risk assessment. This approach offers practical insights for developing digital surveillance and early warning systems in public health.

## Introduction

Hand, foot, and mouth disease (HFMD), an infectious disease primarily affecting children younger than 5 years, has emerged as an important global public health challenge [1,2]. Driven mainly by enterovirus 71 (EV71) and coxsackievirus A16 (CA16), HFMD is easily spread in environments where children congregate [3]. It’s typically 3‐5 days of incubation that precedes distinctive rashes and vesicles on the hands, feet, and oral cavity [4]. The Asia-Pacific region bears a high burden [5], with China reporting a record of 1.68 million cases in 2023 [6]. For timely and targeted interventions, understanding the circulation mechanisms of HFMD and an accurate prediction model for real future risk assessment are needed.

The epidemiology of HFMD is shaped by a complex interplay of systematic factors. Beyond pathogen and host immunity impacts, meteorological conditions and air quality emerge as critical determinants of HFMD transmission dynamics [7]. A growing body of evidence highlights the significant influence of temperature, relative humidity, wind speed, and diurnal temperature range (DTR) on HFMD transmission [8-12]. In addition, air pollution has emerged as a crucial cofactor, with PM_2.5_, PM_10_, SO_2_, NO_2_, and O_3_ levels demonstrating significant associations with HFMD incidence [9,10,13,14]. These environmental factors likely influence HFMD transmission through multiple pathways: altering viral viability, modulating host immunity, and affecting human behavior patterns [15,16]. Consequently, when constructing a risk assessment framework for HFMD, meteorological factors and air pollution should be considered as essential components.

While environmental drivers are well documented, traditional surveillance systems (eg, case reports) struggle to capture real-time transmission dynamics of HFMD. Web-based search data are a novel tool that can provide real-time insights via public queries on symptoms, treatments, and prevention [17,18]. Numerous studies demonstrate web-based search data’s use in enhancing disease incidence prediction. Incorporating Baidu search index improves HFMD model accuracy [19], while Google Influenza Trends successfully tracked influenza outbreaks [20]. Similar applications in dengue, scarlet fever, chickenpox, and Ebola outbreaks [21-23] further validate this approach. However, challenges remain, including media-driven search distortion, inconsistent web-based penetration affecting data representativeness, and policy-induced shifts in search patterns [24]. Therefore, an optimal HFMD risk assessment framework should integrate traditional surveillance and web-based search data synergistically, balancing their complementary strengths for enhanced accuracy and timeliness.

Advancements in predictive modeling now offer opportunities to leverage these multisource data more effectively. Early studies predominantly used Seasonal Autoregressive Integrated Moving Average (SARIMA) time series models, which effectively capture annual cyclical and seasonal variations but require high data stationarity and struggle with nonlinear relationships and outliers [25-27]. Machine learning introduced advanced algorithms (eg, Extreme Gradient Boosting [XGBoost] and random forest [RF]), outperforming traditional models in capturing complex epidemiological patterns [28,29]. Hybrid models have further enhanced accuracy, such as ARIMA-EEMD-LSTM, combining time series decomposition with neural network adaptability [30-33]. However, current studies lack systematic comparisons between traditional and advanced models’ predictive performance, as well as clear risk-level translation—both critical for accurate HFMD early warning.

Beyond model performance, the ultimate goal of ensuring the effectiveness of an HFMD risk assessment framework depends on its capacity to translate case predictions into actionable risk levels for prevention. Several Chinese cities have developed an influenza index and HFMD index [34-39]. For example, Shenzhen Center for Disease Control and Prevention implemented an HFMD risk index to guide targeted prevention measures based on risk levels [36,37], advising improved hygiene and avoidance of crowded areas during high-risk periods. However, current HFMD risk assessments often depend on single-source surveillance data for short-term (1-week ahead) predictions, limiting accuracy. Although some studies have developed multisource prediction models, effectively translating forecasts into actionable risk levels remains rare. This gap highlights the need for a more comprehensive risk assessment framework integrating multisource data for early warning and effective prevention.

This study is conducted in Bao’an District of Shenzhen, a typical high-density subtropical urban region within the Guangdong-Hong Kong-Macao Greater Bay Area that experiences persistent challenges in HFMD transmission. Despite the high disease burden, prediction research integrating environmental drivers with epidemiological patterns has remained limited. To address this gap, this study aims to develop a comprehensive HFMD risk assessment framework by integrating multisource data (historical incidence, environmental parameters, and web-based search behavior data), enabling the translation of predicted cases into actionable risk levels to support prevention strategies (Figure S1 in Multimedia Appendix 1).

## Methods

### Study Site

Bao’an District, located on the eastern bank of the Pearl River Estuary in the northwest of Shenzhen, a coastal region in South China, spans a total area of 724.6 square kilometers. The district’s permanent resident population reached 4.5654 million in 2023, ranking first in the city. Characterized by a subtropical monsoon maritime climate, Bao’an District enjoys ample sunlight, abundant rainfall, and a mild climate. Specifically, the region has an average annual temperature of 22 °C, an average relative humidity of 76%, and an air quality excellence rate exceeding 90%. Regarding environmental air quality, particulate matter and ozone are the primary pollutants.

### Data Sources

#### Incidence Data

The first dataset of this study comprises the case count of HFMD. We collected case data for Bao’an District, Shenzhen, from January 1, 2014, to December 31, 2023, through the “China Information System for Disease Control and Prevention.” HFMD is classified as a Category C notifiable infectious disease, with case reporting adhering to the regulations of the “Law of the People’s Republic of China on the Prevention and Treatment of Infectious Diseases” and the “Norms for the Management of Infectious Disease Information Reporting.” The diagnostic criteria adhered to the Health Industry Standards of the People’s Republic of China (WS 588‐2018) [40]. The collected information documented the patient’s gender, date of birth, and date of symptom onset. In this study, the daily number of HFMD cases was calculated based on the date of symptom onset.

#### Environment Data

Meteorological data were collected from an online platform that provides global weather information, encompassing daily average temperature, maximum temperature, minimum temperature, relative humidity, wind speed, and atmospheric pressure in Shenzhen. In addition, the DTR was calculated as the difference between daily maximum and minimum temperatures. Air pollution data were obtained from the Qingyue Data website, which includes daily average concentrations of fine particulate matter (PM_2.5_, μg/m^3^), inhalable particulate matter (PM_10_, μg/m^3^), sulfur dioxide (SO_2_, μg/m^3^), nitrogen dioxide (NO_2_, μg/m^3^), carbon monoxide (CO, mg/m^3^), and ozone (O_3_, μg/m^3^) in Shenzhen.

#### Baidu Index

In this study, we collected daily HFMD-related keywords (n=51) from a commercial website and Baidu Index and supplemented them with additional terms (n=5) based on prior literature and etiological knowledge [41]. After excluding duplicates (n=2) and terms with a correlation coefficient less than 0.4 with HFMD (n=33), a total of 22 terms remained. These terms were categorized into 4 groups: definition (n=6), symptom (n=6), treatment (n=7), and prevention (n=2) (Table S1 in Multimedia Appendix 2). To construct 4 separate composite Baidu Indexes corresponding to each category, we calculated the product of each term’s search frequency and its correlation coefficient with HFMD, normalized this product by the total correlation coefficient to obtain a ratio, and then summed these ratios within each category. These composite indexes were used to analyze and predict HFMD incidence trends.


(1) $$CI=\sum \left(N_{i}\times r_{i}\right)/\sum r_{i}$$

In equation (1), CI denotes the composite index, a metric designed to quantify the relationship between search trends of specific Baidu Index–related keywords and HFMD case numbers. *N*_*i*_ represents the search index for the *i* th keyword, reflecting the search volume of that HFMD-related keyword. *r*_*i*_ is Pearson correlation coefficient between the search index of the *i* th keyword and HFMD case numbers. $\sum r_{i}$ denotes the sum of Pearson correlation coefficients for all related keywords, serving as a normalizing factor to account for the collective impact of all selected keywords in the composite index.

#### Public Health and Social Measures

The Government Response Indicator was obtained from the COVID-19 Government Response Tracker . This composite index, based on 13 policy response indicators such as school closures, workplace closures, travel bans, testing policies, contact tracing, mask mandates, and vaccination policies, is standardized on a scale of 0-100, reliably measuring the intensity of public health and social measures (PHSMs) over time.

### Statistical Analysis

#### Descriptive Analysis

Descriptive statistics were used to characterize HFMD case distributions across population, temporal, and spatial dimensions, and to assess the distribution of environmental factors and PHSMs. Spearman correlation analysis was used to evaluate the associations between HFMD case counts and both environmental factors and PHSMs. In addition, Pearson correlation coefficient was used to assess the correlation between the Baidu Index and the number of HFMD cases.

We used the Partial Autocorrelation Function (PACF) to analyze the autocorrelation between HFMD incidence and its lagged values. The PACF measures the direct correlation between the current value *y*_t_ and the lagged value *y*_t-k_, after removing the effects of intermediate lags (*y*_t-1_, *y*_t-2_,..., *y*_t-k-1_) [42]. This analysis helped identify significant lagged correlations in the HFMD incidence time series, which informed model selection and parameter estimation.

We conducted lagged cross-correlation analysis to assess the relationship between Baidu Index and HFMD incidence at various time lags. This method evaluates the correlation between 2 time series while considering the dynamic interactions over time [43]. By calculating cross-correlation coefficients at different lags, we identified potential time-lagged associations between web-based search data and HFMD incidence. These findings provided essential time series feature information for constructing predictive models.

#### Factor Analysis

This study used the Distributed Lag Nonlinear Model (DLNM) to analyze the risk factors influencing HFMD. DLNM, which focuses on cross-basis functions, is capable of examining nonlinear and lagged effects between exposure and response variables. It has been widely used to study the complex impacts of environmental exposures on diseases [44]. By integrating the results of Spearman correlation analysis and excluding collinearity among environmental indicators, we ultimately selected environmental factors associated with HFMD for inclusion in the model. Environmental factors were treated as independent variables, with weekly case counts as the dependent variable, while controlling for confounding factors such as day-of-week effects, holiday effects, and long-term trends. Allowing for overdispersion, a quasi-Poisson regression was applied based on DLNM. The model formula is as follows:


(2) $$Y_{t}∼Quasi-Poisson\left(\mu_{t}\right)$$

(3) $$\begin{array}{l} log\;E[\mu_{t}]=\alpha +cb(var)+ns({time}_{t},df=7/year)\\ +\beta {DOW}_{t}+\gamma Holiday_{t}+f({cov}_{i})+{autoregressive}_{terms} \end{array}$$

In equations(2)  (3), *Y*_*t*_ represents the number of HFMD cases in week *t*; *α* represents the intercept; and var refers to meteorological factors or air pollutants. The function cb stands for cross-basis that integrates both exposure and lag dimensions. Dow_*t*_ represents the day-of-week effect, and Holiday_*t*_ represents the holiday effect. ns indicates natural spline functions, and time_*t*_ controls for long-term trends. The function *f* (cov) refers to other factors except var in the model to control the confounding effect. Autoregressive terms refer to the autoregressive terms of daily HFMD counts [45]. We used a smoothing function to manage the first and second lags of the number of cases in our model, indirectly reflecting the effect of population immunity on the pattern of HFMD transmission. The maximum lag days and degrees of freedom in the model were determined using the Akaike information criterion for quasi-Poisson (Q-AIC). The maximum lag time for environmental factors was set to 14 days, and the long-term trend was set to 7 per year. In this model, both environment factors and lag spaces were fitted using natural cubic spline functions, with 4 degrees of freedom (*df*) for environment factors and 3 *df* for lag spaces, based on the Q-AIC and prior literature [10].

#### Prediction Model

The prediction task was formulated as a retrospective time series regression problem, with the target being the weekly number of HFMD cases in the subsequent 1-4 weeks (Figure S1 in Multimedia Appendix 3). Given that previous studies have demonstrated the high accuracy of the SARIMA model in short-term forecasting of HFMD, we first constructed a prediction model using only HFMD case counts, based on the SARIMA model (Table S1 in Multimedia Appendix 4).

We then developed 1- to 4-week-ahead predictive models using 3 tree-based machine learning algorithms: RF, XGBoost, and Light Gradient Boosting Machine (LightGBM). Predictor variables included environmental factors associated with HFMD identified in previous analyses, Baidu Composite Index, PHSMs, holidays, week numbers, and historical HFMD case counts reflecting infection sources and population immunity (Table S1 in Multimedia Appendix 4). To address multicollinearity, variables with pairwise correlation coefficients exceeding 0.8 were screened, and those less correlated with HFMD incidence were excluded.

To further enhance prediction performance, we used a stacking ensemble strategy. The 3 tree-based models served as base learners, and their prediction outputs were used as input features for a metalearner. To reduce the risk of overfitting, we selected a linear regression model as the metalearner due to its simplicity and strong generalization ability. The final ensemble model was trained using this 2-level architecture.

Data from 2014 to 2023 were divided into a training set (2014‐2022) and a testing set (2023). Model hyperparameters were tuned using 5-fold time series cross-validation on the training set. Based on the optimized parameters, we constructed 1- to 4-week-ahead predictive models for HFMD incidence using each of the 3 base models (RF, XGBoost, and LightGBM) as well as the stacking ensemble model.

Model performance was evaluated on the testing set using several common regression metrics, including the coefficient of determination (*R*²), Pearson correlation coefficient (*r*), mean absolute error (MAE), and root-mean-square error (RMSE). Lower MAE, lower RMSE, higher *R*^2^, and higher *r* indicated better forecasting performance (Formula 1 in Multimedia Appendix 5).

#### Risk Assessment

Based on the weekly HFMD case data from 2014 to 2019 (before COVID-19 pandemic), the cumulative distribution function of weekly case counts was calculated. The 40th, 60th, and 80th percentiles of the cumulative distribution function were then used as thresholds to classify epidemic risk levels. The selection of the 40%, 60%, and 80% quantiles as risk thresholds was based on 2 considerations. First, local experts recommended quantile-based thresholds for their practicality in supporting tiered risk management. Second, similar percentile-based classifications have been adopted in previous studies [34,39], which informed our framework design. Specifically, the predicted weekly case counts were compared against the aforementioned thresholds to determine the risk level as follows. High risk: case count >80th percentile; moderate risk: 60th percentile < case count ≤ 80th percentile; medium risk: 40th percentile < case count ≤ 60th percentile; and low risk: case count ≤ 40th percentile.

The accuracy of risk assessment was evaluated by calculating the accuracy rate, overestimation rate, and underestimation rate (Formula 2 in Multimedia Appendix 6). Higher accuracy and lower overestimation and underestimation rates indicated better risk assessment performance.

#### Software and Visualization

Data cleaning and descriptive analysis, as well as predictive analysis, were performed using Python (version 3.12.4; Python Software Foundation). Predictive models included SARIMA (pmdarima), machine learning algorithms such as RF (sklearn), extreme gradient boosting (xgboost), light gradient boosting (lightgbm), and a stacked model constructed using sklearn components. The DLNM was constructed in R (version 4.4.2; R Foundation for Statistical Computing) using the dlnm package.

### Ethical Considerations

All data used in our study was anonymized and deidentified and did not involve data related to humans. Therefore, our research was exempted from the requirement of written informed consent and was approved by the ethics committee of the Bao’an Center for Disease Control and Prevention.

## Results

### Data Description

**Figure 1.**
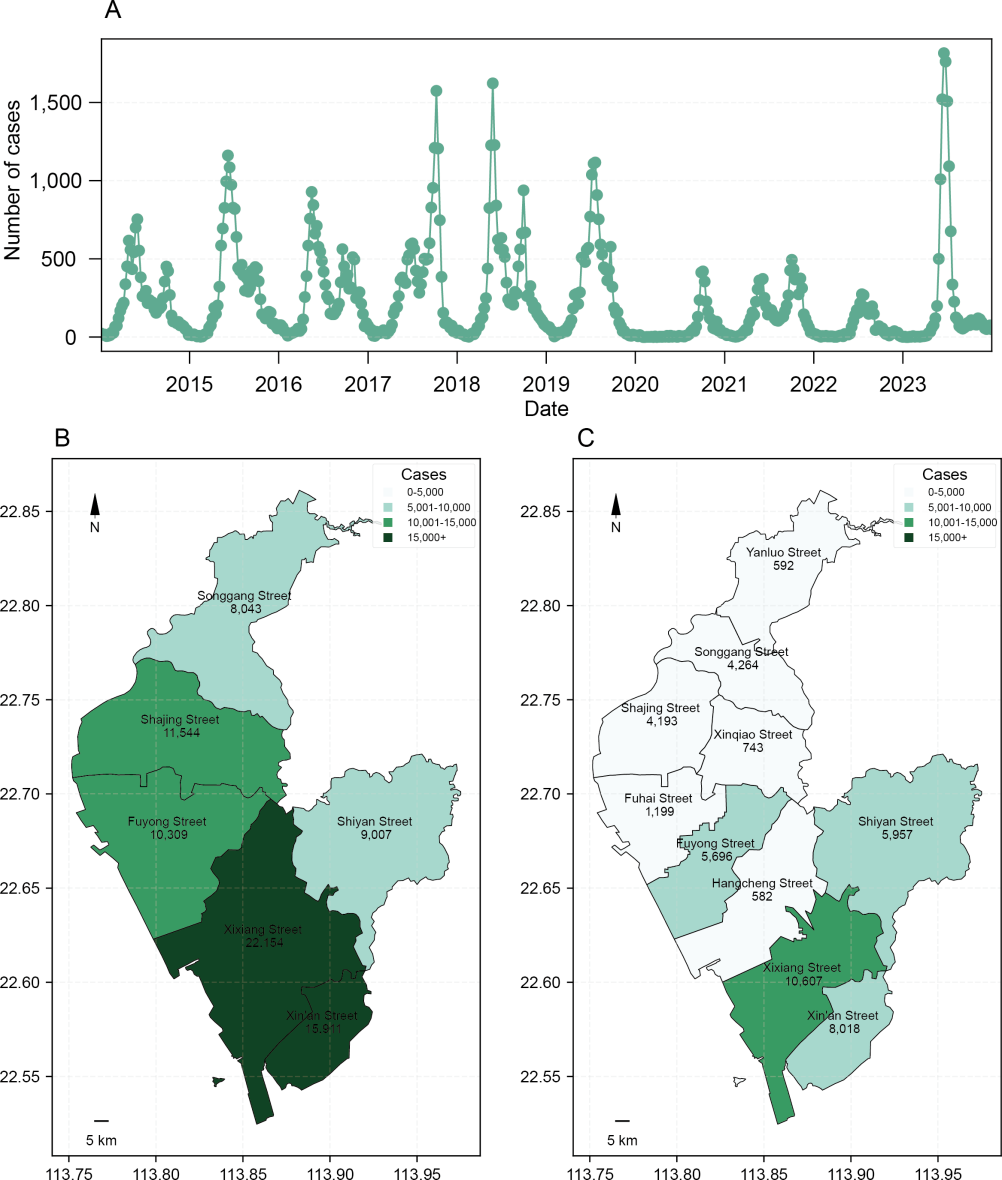
Temporal and spatial distribution of incidence of hand, foot, and mouth disease (HFMD) in Bao’an District from 2014 to 2023. (A) Time series diagram of the number of weekly HFMD cases. (B) Spatial distribution of the number of HFMD cases in Bao’an District from 2014 to 2018. (C) Spatial distribution of the number of HFMD cases in Bao’an District from 2019 to 2023.

From 2014 to 2023, Bao’an District in Shenzhen City reported a total of 118,826 HFMD cases, 93.6% (111,181/118,826) of which occurred in children aged 5 years and younger (Table 1). The incidence of HFMD displayed a significant seasonal pattern characterized by a bimodal distribution (Figure 1A). The primary peak spanned from approximately week 12 to week 32, coinciding with the spring and summer seasons, while a secondary peak emerged from around week 36 to week 45, corresponding to the autumn and winter seasons (Figure 1A). During the study period, the incidence of HFMD in Bao’an District exhibited significant geographical variations. The highest incidence was observed in the central areas, specifically in Xin’an and Xi’xiang subdistricts (Figure 1B and C).

**Table 1.** Characteristics of the hand, foot, and mouth disease cases in Bao’an district of Shenzhen from 2014 to 2023.

Characteristics	Proportion, n (%)
Sex	
Male	71,441 (60.1)
Female	47,385 (39.9)
Age (years)	
0‐5	111,181 (93.6)
6‐12	6455 (5.4)
13‐15	147 (0.1)
16‐18	65 (0.1)
19‐60	972 (0.8)
>60	6 (0.0)
Career	
Children in the diaspora	88,858 (74.8)
Children in nursery	24,809 (20.9)
Students	4169 (3.5)
Others	990 (0.8)

Throughout the study period, meteorological factors such as temperature, atmospheric pressure, relative humidity, DTR, and air pollutants such as PM_2.5_, PM_10_, SO_2_, NO_2_, O_3_, and CO, as well as Baidu Index showed obvious seasonal fluctuations (Figure S1 in Multimedia Appendix 7 and Figure S1 in Multimedia Appendix 8).

### Systematic Factors Affecting HFMD

Table S1 in Multimedia Appendix 9 provides an overview of the statistical distribution of HFMD case counts and systematic factors. During the study period, the average number of HFMD cases was 32.53. Spearman correlation analysis between HFMD and environmental factors (Table S1 in Multimedia Appendix 9) revealed that daily average temperature exhibited the most significant correlation with HFMD, with a correlation coefficient of 0.62. Except for SO_2_, HFMD showed negative correlations with other air pollutants.

PACF analysis (Figure S1 in Multimedia Appendix 10) revealed significant autocorrelation between HFMD incidence and its lagged values at 1- to 3-week intervals. Lagged cross-correlation analysis (Figure S1 in Multimedia Appendix 11) indicated that Baidu search index, including both the composite index and the sub-index for definition, symptoms, treatment, and prevention, exhibited the strongest correlation with HFMD case counts during the week of disease onset.

**Figure 2.**
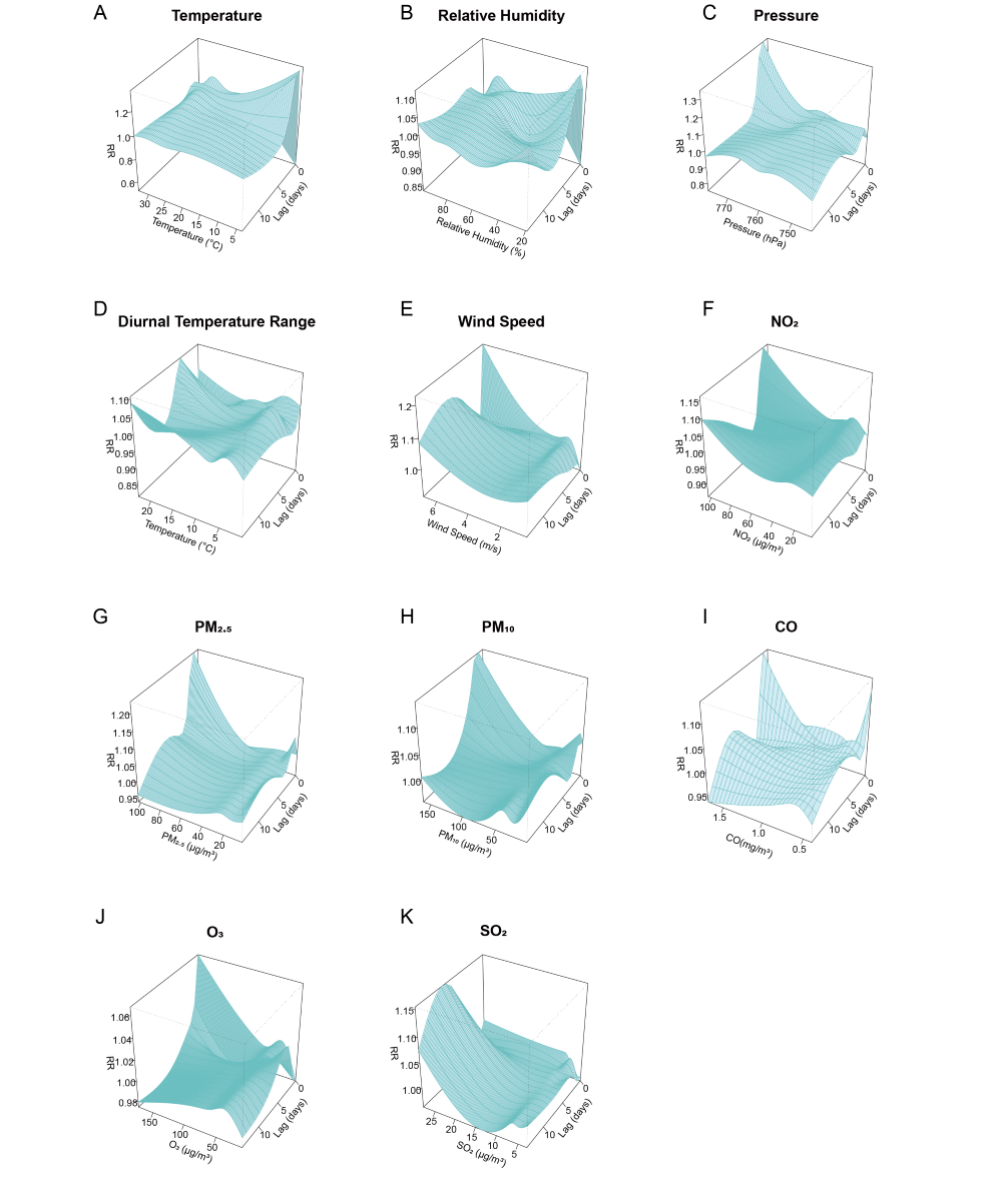
Relative risks for hand, foot, and mouth disease incidence associated with environmental factors and lags using Distributed Lag Nonlinear Models in Bao’an District from 2014 to 2023: (A) temperature, (B) relative humidity, (C) air pressure, (D) daily temperature range, (E) wind speed, (F) NO_2_, (G) PM_2.5_, (H) PM_10_, (I) CO, (J) O_3_, and (K) SO_2_.

The analysis of influencing factors indicates that meteorological variables and air pollutants exhibit lagged and nonlinear effects on HFMD incidence (Figure 2 and Figure S1 in Multimedia Appendix 12). Temperature demonstrates an inverted V-shaped relationship with relative risk increasing up to a specific threshold before declining (Figure 2A). Low relative humidity appears to have a protective effect, whereas low atmospheric pressure and a reduced DTR are associated with an elevated risk of HFMD (Figure 2B-D). Wind speed follows an S-shaped pattern in its association with HFMD incidence (Figure 2E). In our analysis, ambient concentrations of PM_2.5_, PM_10_, CO, and O_3_ were inversely associated with HFMD risk (Figure 2G-J), with higher pollutant levels correlating with a lower relative risk of disease. Furthermore, NO_2_ demonstrated a positive association with HFMD incidence at low concentration ranges (Figure 2F), while its effect was not statistically significant at higher or extremely low concentrations. In addition, SO_2_ does not show a statistically significant impact on HFMD incidence and was therefore excluded from subsequent predictive modeling (Figure 2K).

### Predictive Results

**Figure 3.**
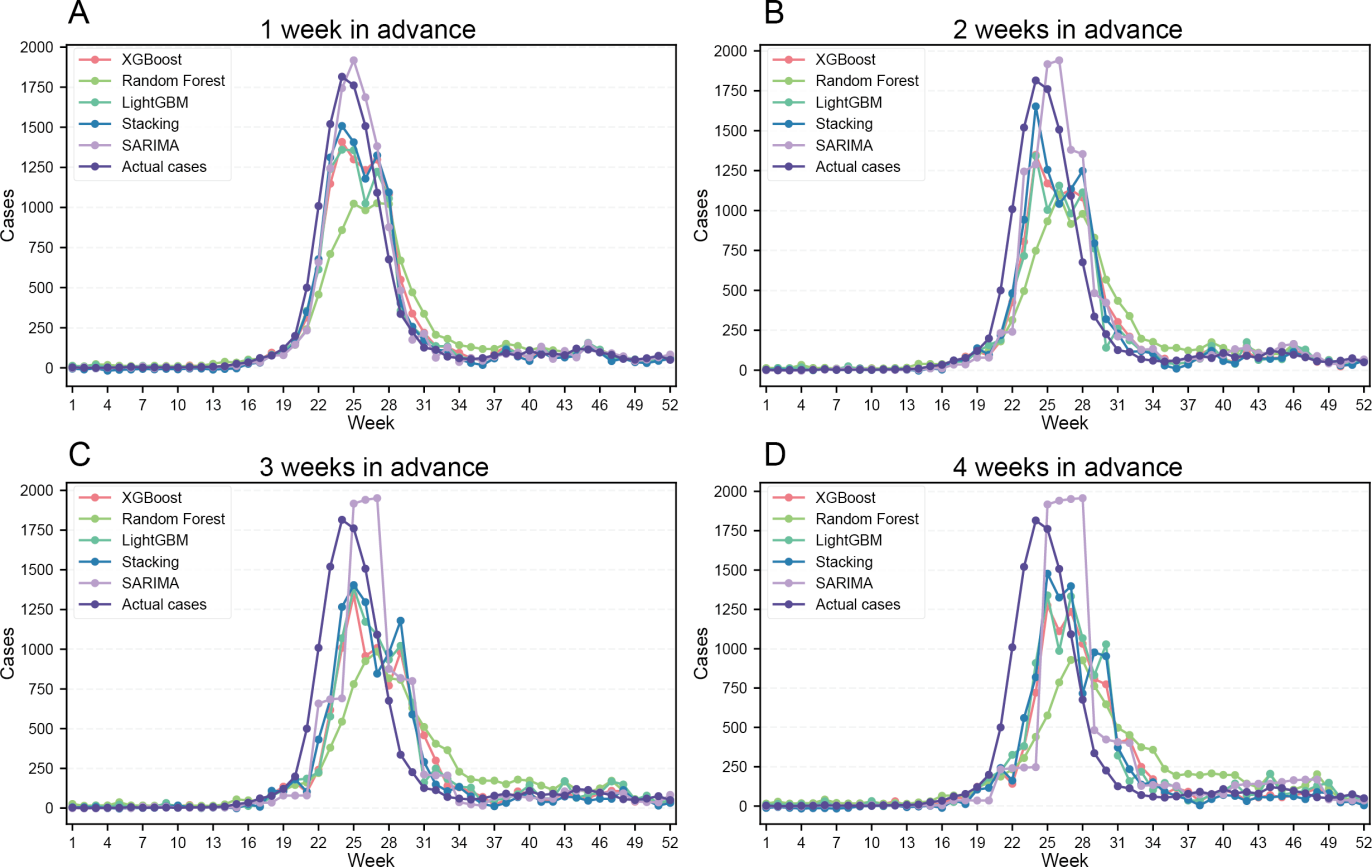
Time series comparison of predicted and observed values by different models for 1- to 4-week-ahead forecasts on the 2023 test set. (A) 1-week-ahead prediction versus observed. (B) 2-week-ahead prediction versus observed. (C) 3-week-ahead prediction versus observed. (D) 4-week-ahead prediction versus observed. LightGBM: Light Gradient Boosting Machine; SARIMA: Seasonal Autoregressive Integrated Moving Average; XGBoost: Extreme Gradient Boosting.

The prediction results indicate that the SARIMA model exhibited the highest predictive accuracy for the 1-week-ahead forecast (*R*²=0.95, *r*=0.98, MAE=53.34, and RMSE=99.31) (Table S1 in Multimedia Appendix 13 and Figure 3A). For midterm forecasts (2‐4 weeks ahead), the ensemble model integrating multiple machine learning algorithms demonstrated superior performance (2 weeks: *R*²=0.83, *r*=0.92, MAE=87.84, and RMSE=185.08; 3 weeks: *R*²=0.75, *r*=0.87, MAE=112.41, and RMSE=229.13; and 4 weeks: *R*²=0.64, *r*=0.80, MAE=132.47, and RMSE=276.81) (Table S1 in Multimedia Appendix 13 and Figure 3B-D).

Moreover, the predicted incidence curves closely aligned with the observed epidemic trends, further supporting the reliability of the models (Figure 3). However, some temporal discrepancies were observed in peak incidence predictions, with slight time lags in forecasting epidemic peaks (Figure 3). In addition, the predictive accuracy exhibited a gradual decline as the forecasting horizon extended from 1 to 4 weeks (Figure 3).

### Risk Assessment Index

**Figure 4.**
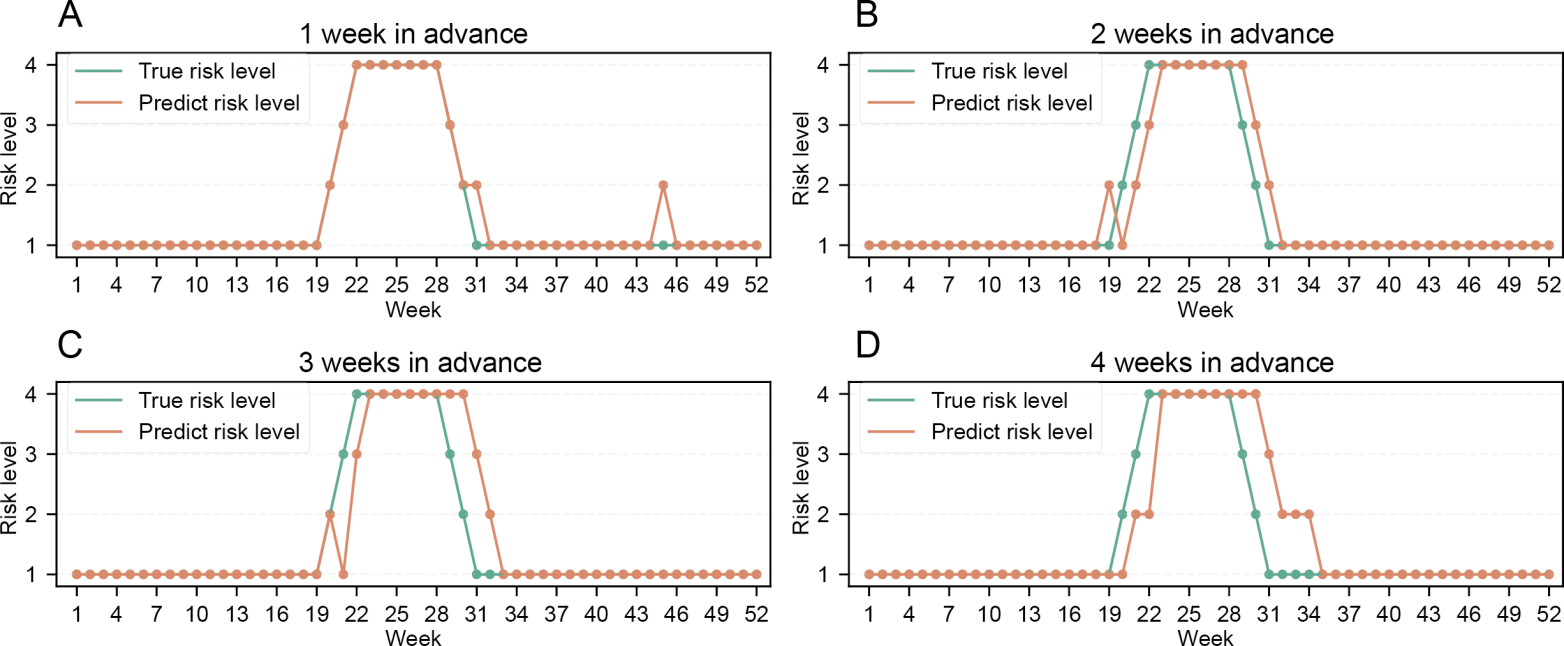
The results of risk levels of hand, foot, and mouth disease (HFMD) for 1-4 weeks ahead using stacking models. (A) 1-week-ahead HFMD risk levels. (B) 2-week-ahead HFMD risk levels. (C) 3-week-ahead HFMD risk levels. (D) 4-week-ahead HFMD risk levels.

The risk assessment analysis for HFMD in Bao’an District during 2023, based on the prediction results of the stacking model, demonstrated good predictive performance (Table S1 in Multimedia Appendix 14 and Figure 4A-D), with forecast accuracy exceeding 80% across all 1- to 4-week-ahead prediction windows. The 1-week-ahead short-term prediction model achieved exceptional accuracy with forecast accuracy of 96% (Figure 4A and Table S1 in Multimedia Appendix 14).

## Discussion

### Principal Findings

Based on a comprehensive analysis of the impact of systemic factors on HFMD, we innovatively designed an HFMD risk prediction framework by comparing traditional and advanced machine learning prediction models. Our findings suggest that air pollutants and meteorological factors other than SO₂ have a significant effect on the incidence of HFMD (Figure 2 and Figure S1 in Multimedia Appendix 12). In addition, Baidu Index proves to be an effective tool for capturing the epidemic trend of HFMD during the onset week (Figure S1 in Multimedia Appendix 11). While the SARIMA model performs well in 1-week-ahead short-term forecast, advanced machine learning methods incorporating systematical factors performed better for 2- to 4-week-ahead midterm forecasts (Table S1 in Multimedia Appendix 13 and Figure 3). Furthermore, the predicted risk levels based on the advanced forecast models aligned closely with the actual levels (Table S1 in Multimedia Appendix 14 and Figure 4).

Our analysis revealed that meteorological factors exert nonlinear effects on HFMD incidence (Figure 2 and Figure S1 in Multimedia Appendix 12). Temperature exhibits an inverted V-shaped effect on the risk of HFMD, corresponding to optimal conditions for enterovirus survival and transmission [8,9]. Elevated relative humidity enhances HFMD transmission, likely through prolonged virus stability in aerosols [10-12]. Wind speed exerts dual effects: moderate levels reduce risk via particle dispersion [11,46], while extreme winds may increase transmission through environmental disruption. The daily temperature variation and other influences of atmospheric pressure indicate that there is a complex interaction between climate and pathogens, but the mechanism details need further study [8].

Our findings showed a negative nonlinear association between ambient concentrations of PM₂.₅, PM₁₀, O₃, and CO and HFMD incidence, consistent with evidence that ozone’s virucidal oxidative effects and pollution-induced behavioral changes (eg, reduced outdoor activity) may suppress transmission [9,10,13,14]. Our study found that low-concentration NO_2_ exposure exhibited a positive association, likely mediated by NO_2_-induced respiratory inflammation and impaired mucosal defenses [10]. The effects diminished beyond a narrow exposure range, indicating threshold-dependent influences. In contrast, SO_2_ showed no statistically significant relationship with HFMD incidence, possibly due to regional differences in emissions, atmospheric chemistry, or population susceptibility. Collectively, these findings illustrate the nonlinear relationship between multidimensional environmental exposures and HFMD transmission patterns. These insights informed the development of our multifactorial prediction framework, which improved predictive accuracy.

This study also evaluated the predictive value of web-based search data for the incidence of HFMD. Our results show that the composite Baidu Index and its subindex are effective in capturing epidemiological fluctuations in the week of onset (Figure S1 in Multimedia Appendix 11), which is consistent with previous findings [19]. Compared with traditional passive monitoring that relies on laboratory confirmations and case reports, Baidu search data have the advantage of real-time performance and can reflect epidemic changes 3-7 days in advance, which provides a key supplement for early warning. Similar to previous studies [17,19], this study constructed a multidimensional Baidu comprehensive index by screening core subindicators. This method not only improves the prediction accuracy but also realizes the dynamic tracking and trend prediction of public health concerns by integrating multidimensional search data (rather than a single indicator). It proves the dual advantages of Baidu Index in infectious disease surveillance, which is both timely and comprehensive. These findings not only verify the universality of digital epidemiology in local areas but also provide a paradigm for other low-income countries to optimize infectious disease surveillance by using localized web-based data.

Our study further compared the predictive performance of traditional models and advanced machine learning models on different time scales. The results showed that SARIMA demonstrated higher accuracy in 1-week-ahead short-term forecast than other machine learning models, which is consistent with previous studies in Nanjing [26] and Sabah [27]. The advanced machine learning models exhibited superior predictive capacity for 2- to 4-week-ahead midterm forecasts (Table S1 in Multimedia Appendix 13 and Figure 3). Our findings align partially with a Japanese Long Short-Term Memory Network–based study [29] but achieve earlier detection (2‐4 weeks) and higher accuracy through multimodel integration. Unlike prior hybrid model studies in Wuhan [33], Chongqing [30], and Xinjiang [32], our multiscale framework uniquely combines predictive performance with optimized model selection across temporal scales, offering more actionable guidance. SARIMA is optimal for rapid response scenarios requiring immediate decisions, as it relies solely on historical incidence data without requiring additional variables [31,33]. Machine learning better supports midterm preparedness planning due to its ability to incorporate diverse predictors, although it demands greater technical capacity. For practical implementation, we recommend aligning model selection with both operational timelines (short-term vs midterm needs) and local data infrastructure, while future research should explore hybrid systems that combine SARIMA’s reliability with machine learning’s adaptability, alongside translating forecasts into operational risk assessments.

In recent years, the importance of infectious disease risk prediction in disease control has increased. Risk indices for common infectious diseases such as influenza and HFMD have been developed in a number of cities in China, including Shenzhen [37], Zhuhai [35], Beijing [39], and Maanshan [38]. These indices are released to the public to provide early warning of disease risks, thereby raising public health awareness and promoting healthy behaviors with positive social benefits. However, existing risk indices are largely based on notifiable infectious disease reports and sentinel hospital surveillance data, with insufficient consideration of the impact of systemic factors on disease transmission. Moreover, prediction models often rely on simple multiple linear equations, resulting in suboptimal prediction accuracy. For instance, the predicted concordance rate of the HFMD risk index in Shenzhen was only 77.8% between August 2017 and November 2018 [36]. To overcome previous limitations, this study integrates diverse data sources with advanced machine learning models, developing a more accurate and reliable HFMD risk prediction framework (risk-level accuracy of >90%; Figure 4 and Table S1 in Multimedia Appendix 14). Operationalized in Shenzhen’s Bao’an District, our model outperforms existing methods by incorporating multidimensional environmental and epidemiological data to deliver precise 1‐ to 4-week forecasts. This provides a robust, data-driven foundation for public health decision-making and proactive community guidance.

### Limitations

This study acknowledges several limitations. First, the study was conducted in a limited geographic area, which may restrict the generalizability of the findings. Second, we used average exposure estimates for meteorological factors and air pollutants in Shenzhen rather than individual direct measurement, resulting in exposure measurement errors, but they are likely to be random and nondifferential. Third, the HFMD surveillance data were obtained from a passive monitoring system, which inherently fails to capture all cases, particularly those with mild symptoms that do not warrant medical consultation. Fourth, the transmission of HFMD is influenced by an even more extensive range of factors, including individual vaccination and immune status, lifestyle practices, hygiene practices, contact patterns (particularly in households and childcare settings), indoor environments, ventilation conditions, socioeconomic determinants, and population metrics, such as size and density. Additionally, different types of pathogens may affect the transmission patterns of HFMD, highlighting the importance of laboratory-based pathogen data analysis for identifying the temporal trends and characteristics of HFMD. Finally, with the rapid advancement of artificial intelligence technologies, exploring their effective application in HFMD prediction is an important direction for future research. Future studies should further investigate the roles of these multidimensional factors and integrate more advanced predictive techniques to better understand and monitor the epidemiological trends of HFMD, thereby effectively reducing the risk of infection.

### Conclusions

With explorations of the complex influencing pattern for systematic factors, this study developed a prediction model and future epidemic risk assessment framework for HFMD by integrating HFMD incidence data, environmental factors, Baidu Index, and public health interventions using advanced machine learning algorithms. The results highlight the significant role of systematic factors in long-term HFMD predictions and precise risk assessment and demonstrate the model’s potential to enhance public health decision-making. Future research should incorporate additional multidimensional factors, including host characteristics, pathogen properties, and socioeconomic conditions, and further explore their interactive effects with more advanced technologies to optimize HFMD risk prediction and control strategies.

## Supplementary material

10.2196/75434 Multimedia Appendix 1 Research design of the study.

10.2196/75434 Multimedia Appendix 2 Pearson correlation analysis between Baidu search terms and the number of hand, foot, and mouth disease cases in Bao’an District.

10.2196/75434 Multimedia Appendix 3 Flowchart of the predictive model.

10.2196/75434 Multimedia Appendix 4 Features included in the algorithm.

10.2196/75434 Multimedia Appendix 5 Model performance.

10.2196/75434 Multimedia Appendix 6 The accuracy of risk assessment.

10.2196/75434 Multimedia Appendix 7 The temporal distribution of daily hand, foot, and mouth disease cases and environmental factors in Bao’an district of Shenzhen from 2014 to 2023.

10.2196/75434 Multimedia Appendix 8 The temporal distribution of weekly cases and Baidu Index in Bao’an district of Shenzhen from 2014 to 2023.

10.2196/75434 Multimedia Appendix 9 Descriptive statistics of the hand, foot, and mouth disease cases and systematic factors from 2014 to 2023.

10.2196/75434 Multimedia Appendix 10 Autocorrelation plot of weekly number of hand, foot, and mouth disease cases.

10.2196/75434 Multimedia Appendix 11 Cross-correlation coefficients between weekly number of hand, foot, and mouth disease cases and different groups of Baidu Index composite terms.

10.2196/75434 Multimedia Appendix 12 Cumulative lag effect plots of the impacts of environmental factors on the risk of hand, foot, and mouth disease.

10.2196/75434 Multimedia Appendix 13 Evaluation metrics for Seasonal Autoregressive Integrated Moving Average model and advanced machine learning methods for 1-week forecasts, 2-week forecasts, 3-week forecasts, and 4-week forecasts for 2023 data.

10.2196/75434 Multimedia Appendix 14 Accuracy evaluation of risk assessment for 1- to 4-week-ahead forecasts in 2023.

## Abbreviations

DLNM: Distributed Lag Nonlinear Model
DTR: diurnal temperature range
HFMD: hand, foot, and mouth disease
LightGBM: Light Gradient Boosting Machine
MAE: mean absolute error
PACF: partial autocorrelation function
PHSM: public health and social measure
RF: random forest
RMSE: root-mean-square error
SARIMA: Seasonal Autoregressive Integrated Moving Average
XGBoost: Extreme Gradient Boosting
