# Supplementary figures and images for: Hand, Foot, and Mouth Disease Risk Prediction in Southern China: Time Series Study Integrating Web-Based Search and Epidemiological Surveillance Data
Source: JMIR Infodemiology. 2025 Oct 9;5:e75434. doi: 10.2196/75434 (PMC12510436; doi:10.2196/75434)

Multimedia Appendix 1


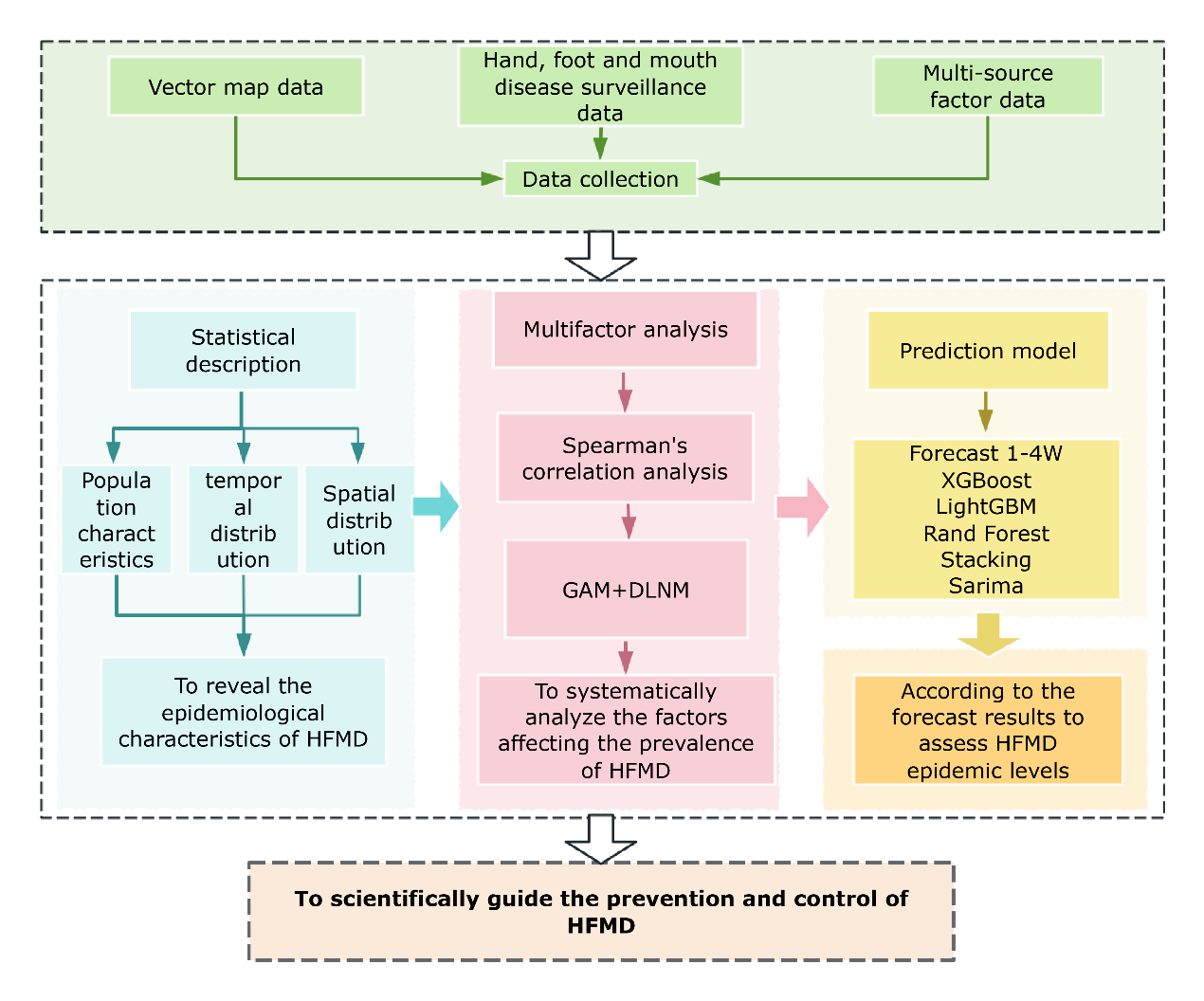


**Figure S1.** Research design of the study.

Supplement: Multimedia Appendix 1 [file infodemiology-v5-e75434-s001.docx]

Multimedia Appendix 3


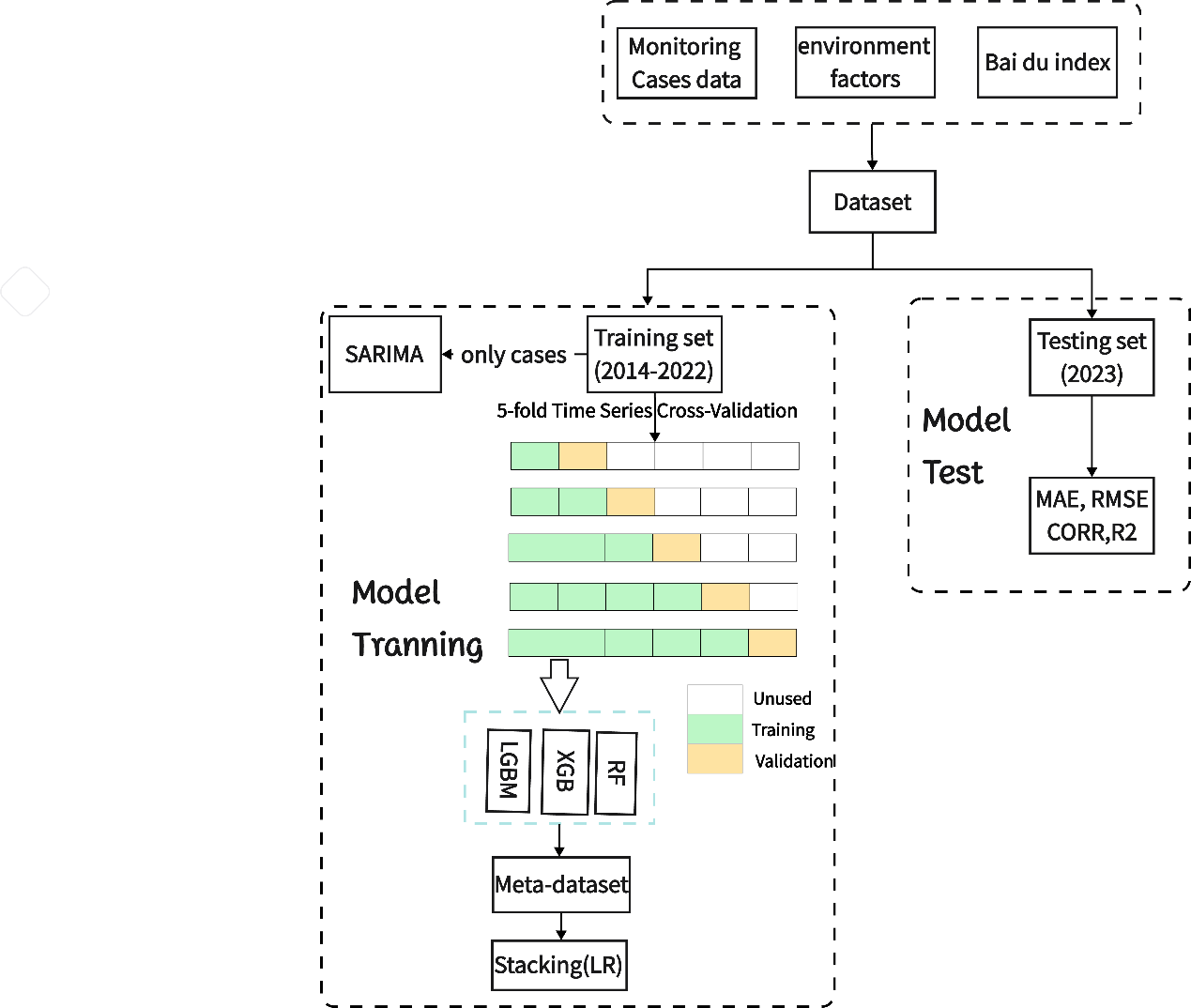


**Figure S1.** Flowchart of the predictive model.

Supplement: Multimedia Appendix 3 [file infodemiology-v5-e75434-s003.docx]

Multimedia Appendix 10


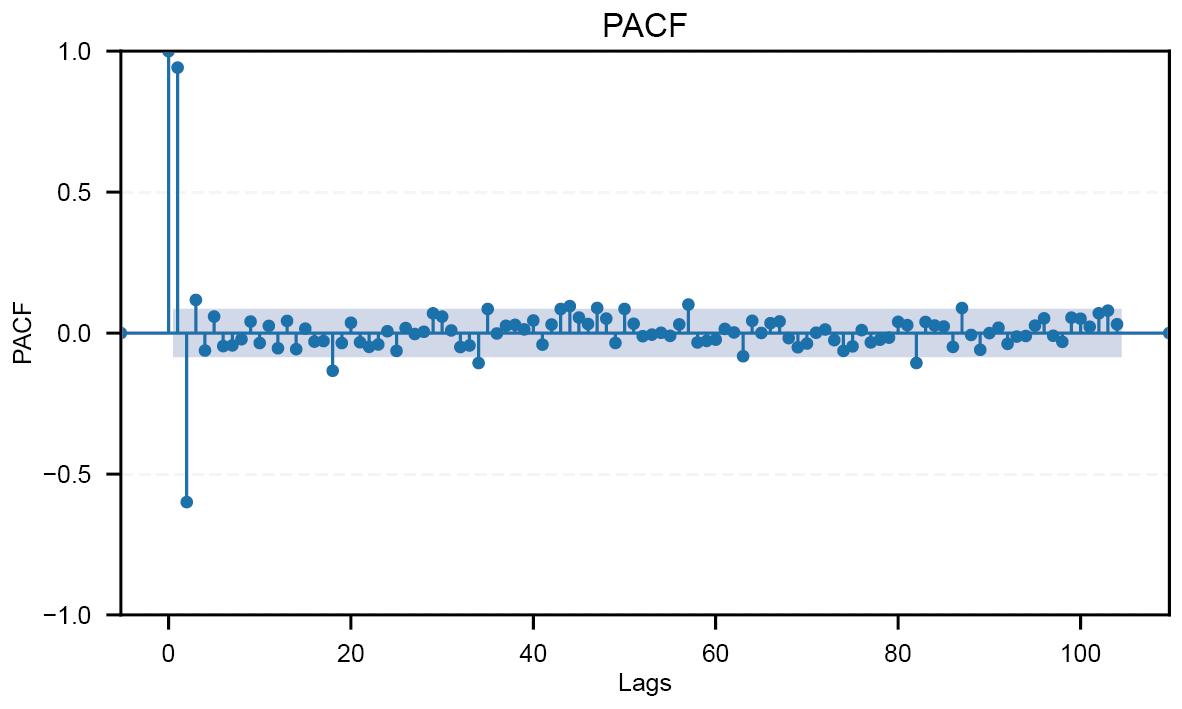


**Figure S1.** Autocorrelation plot of weekly number of HFMD cases.

Supplement: Multimedia Appendix 10 [file infodemiology-v5-e75434-s010.docx]
