# Supplementary material for: Hand, Foot, and Mouth Disease Risk Prediction in Southern China: Time Series Study Integrating Web-Based Search and Epidemiological Surveillance Data
Source: JMIR Infodemiology. 2025 Oct 9;5:e75434. doi: 10.2196/75434 (PMC12510436; doi:10.2196/75434)
Supplement: Multimedia Appendix 2 [file infodemiology-v5-e75434-s002.docx]

Multimedia Appendix 2

Table S1. Pearson correlation analysis between Baidu search terms and the number of HFMD cases in Bao’an District.

| Group | Chinese | English | Pearson correlation coefficient |
| --- | --- | --- | --- |
| Definition | 手足口病 | Hand, Foot, and Mouth Disease (HFMD) | 0.868 |
|  | 手足口 | Hand, Foot, and Mouth | 0.812 |
|  | 手足口病潜伏期 | Incubation period of Hand, Foot, and Mouth Disease | 0.673 |
|  | 儿童手足口病 | Pediatric Hand, Foot, and Mouth Disease | 0.762 |
|  | 什么是手足口病 | What is Hand, Foot, and Mouth Disease? | 0.667 |
|  | 小儿手足口病 | Infantile Hand, Foot, and Mouth Disease | 0.707 |
| Symptom | 小孩手足口病症状 | Symptoms of Hand, Foot, and Mouth Disease in Children | 0.561 |
|  | 手足口病严重吗 | Is Hand, Foot, and Mouth Disease serious? | 0.641 |
|  | 手足口病症状 | Symptoms of Hand, Foot, and Mouth Disease | 0.469 |
|  | 手足口病初期症状 | Early Symptoms of Hand, Foot, and Mouth Disease | 0.575 |
|  | 手足口病图片 | Images of Hand, Foot, and Mouth Disease | 0.683 |
|  | 手足口病有什么症状 | What are the Symptoms of Hand, Foot, and Mouth Disease? | 0.646 |
| Treatment | 手足口病吃什么药 | What Medicine to Take for Hand, Foot, and Mouth Disease? | 0.729 |
|  | 手足口病怎么治疗 | How to Treat Hand, Foot, and Mouth Disease? | 0.705 |
|  | 手足口病治疗 | Treatment for Hand, Foot, and Mouth Disease | 0.700 |
|  | 手足口病用什么药 | What Medicine to Use for Hand, Foot, and Mouth Disease? | 0.730 |
|  | 手足口病疱疹怎么治 | How to Treat Hand, Foot, and Mouth Disease Blisters? | 0.502 |
|  | 手足口病的治疗 | Treatment of Hand, Foot, and Mouth Disease | 0.607 |
|  | 手足口病用药 | Medication for Hand, Foot, and Mouth Disease | 0.673 |
| Prevention | 如何预防手足口病 | How to Prevent Hand, Foot, and Mouth Disease? | 0.589 |
|  | 手足口病预防 | Prevention of Hand, Foot, and Mouth Disease | 0.655 |
