# Supplementary material for: Hand, Foot, and Mouth Disease Risk Prediction in Southern China: Time Series Study Integrating Web-Based Search and Epidemiological Surveillance Data
Source: JMIR Infodemiology. 2025 Oct 9;5:e75434. doi: 10.2196/75434 (PMC12510436; doi:10.2196/75434)
Supplement: Multimedia Appendix 4 [file infodemiology-v5-e75434-s004.docx]

Multimedia Appendix 4

Table S1. Features included in the algorithm.

| Model | Sources of infection and herd immunity | Environmental factors | Internet search index |
| --- | --- | --- | --- |
| SARIMA | HFMD Weekly counts | **/** | **/** |
| XGBoost | HFMD Weekly counts, MD1 ^a^, MD2 ^b^, CumCases_1yr ^c^, CumCases_2yr ^d^ | Weekends and holidays ^e^, WOY ^f^, Temperature, Relative humidity, Wind speed, DTR, PM10, NO2, O3, CO, PHSMs ^g^ | CI_General_Terms ^h^ |
| LightGBM | HFMD Weekly counts, MD1, MD2, CumCases_1yr, CumCases_2yr | Weekends and holidays, WOY, Temperature, Relative humidity, Wind speed, DTR, PM10, NO2, O3, CO, PHSMs | CI_General_Terms |
| RF | HFMD Weekly counts, MD1, MD2, CumCases_1yr, CumCases_2yr | Weekends and holidays, WOY, Temperature, Relative humidity, Wind speed, DTR, PM10, NO2, O3, CO, PHSMs | CI_General_Terms |
| Stacking | HFMD Weekly counts, MD1, MD2, CumCases_1yr, CumCases_2yr | Weekends and holidays, WOY, Temperature, Relative humidity, Wind speed, DTR, PM10, NO2, O3, CO, PHSMs | CI_General_Terms |

^a^ MD1 (First-order Difference): The difference between the number of cases at lag 1 and lag 2, i.e., MD1 = Cases(lag1) - Cases(lag2).

^b^ MD2 (Second-order Difference): The difference between MD1 at lag 1 and MD1 at lag 2, i.e., MD2 = MD1(lag1) - MD1(lag2).

The moving difference between adjacent lags reflects the variation in HFMD cases. Continuous positive and large values of MD1 indicate a rapid increase in cases, signaling a potential outbreak. Large MD2 values suggest an accelerating trend in case growth.

^c^ CumCases_1yr: Cumulative number of cases (CumCases), Total number of HFMD cases accumulated over the past one year.

^d^ CumCases_2yr: Total number of HFMD cases accumulated over the past two years.

^e^ Weekends and holidays: Represents the number of public holidays and weekends in a given week.

^f^ Week of the Year (WOY): Represents the sequential week number within a year (ranging from 1 to 52 or 53, depending on the year).

^g^ Meteorological factors, pollutant levels, and PHSMs were all weekly averages.

^h^ CI_General_Terms: A composite index representing the aggregated search frequency of general HFMD-related terms.
