# Supplementary material for: Hand, Foot, and Mouth Disease Risk Prediction in Southern China: Time Series Study Integrating Web-Based Search and Epidemiological Surveillance Data
Source: JMIR Infodemiology. 2025 Oct 9;5:e75434. doi: 10.2196/75434 (PMC12510436; doi:10.2196/75434)
Supplement: Multimedia Appendix 5 [file infodemiology-v5-e75434-s005.docx]

Multimedia Appendix 5

Formula 1. Model performance.

Model performance was evaluated on the testing set using several common regression metrics, including the coefficient of determination (*R*²), Pearson correlation coefficient (*r*), mean absolute error (MAE), and root mean squared error (RMSE) . Lower MAE, lower RMSE, higher *R*^2^, and higher *r* indicated better forecasting performance.

The coefficients were calculated using the following formulas:

$$R^{2}=1-\frac{\sum_{i=1}^{n} \left( y_{i}-\left[ \hat{y}_{i} \right] \right)^{2}}{\sum_{i=1}^{n} \left( y_{i}-\bar{y}_{i} \right)^{2}}$$

$$\mathrm{MAE}=\frac{1}{n}\sum_{i=1}^{n} \left| y_{i}-\hat{y}_{i} \right|$$

$$\mathrm{RMSE}=\sqrt{\frac{1}{n}\sum_{i=1}^{n} \left( y_{i}-\left[ \hat{y}_{i} \right] \right)^{2}}$$

$$r=\frac{\sum_{i=1}^{n} \left( y_{i}-\bar{y} \right)\left( \hat{y}_{i}-\bar{\hat{y}} \right)}{\sqrt{\sum_{i=1}^{n} \left( y_{i}-\bar{y} \right)^{2}}\sqrt{\sum_{i=1}^{n} \left( \hat{y}_{i}-\bar{\hat{y}} \right)^{2}}}$$

Let $y_{i}$ be the observed (true) value and $\hat{y}_{i}$ be the predicted value for the $i$-th sample, where $n$ is the total number of observations.
