# Supplementary material for: Hand, Foot, and Mouth Disease Risk Prediction in Southern China: Time Series Study Integrating Web-Based Search and Epidemiological Surveillance Data
Source: JMIR Infodemiology. 2025 Oct 9;5:e75434. doi: 10.2196/75434 (PMC12510436; doi:10.2196/75434)
Supplement: Multimedia Appendix 6 [file infodemiology-v5-e75434-s006.docx]

Multimedia Appendix 6

Formula 2. The accuracy of risk assessment.

The accuracy of risk assessment was evaluated by calculating the accuracy rate, overestimation rate, and underestimation rate. Higher accuracy and lower overestimation and underestimation rates indicated better risk assessment performance.

$$\text{Accuracy rate}\text{ (\%)}\text{ = }\frac{N_{\text{correct}}}{N_{\text{total}}}$$

$$\text{Overestimation rate}\text{ (\%)}\text{ =}\frac{N_{\text{over}}}{N_{\text{total}}}$$

$$\text{Underestimation rate}\text{ (\%)}\text{ = }\frac{N_{\text{under}}}{N_{\text{total}}}$$

Where:

$N_{\mathrm{correct}}$: number of weeks where the predicted and observed risk levels are the same

$N_{\mathrm{over}}$: number of weeks where the predicted level is higher than the observed

$N_{\mathrm{under}}$: number of weeks where the predicted level is lower than the observed

$N_{\mathrm{total}}$: total number of weeks
