# Supplementary material for: Hand, Foot, and Mouth Disease Risk Prediction in Southern China: Time Series Study Integrating Web-Based Search and Epidemiological Surveillance Data
Source: JMIR Infodemiology. 2025 Oct 9;5:e75434. doi: 10.2196/75434 (PMC12510436; doi:10.2196/75434)
Supplement: Multimedia Appendix 7 [file infodemiology-v5-e75434-s007.docx]

Multimedia Appendix 7


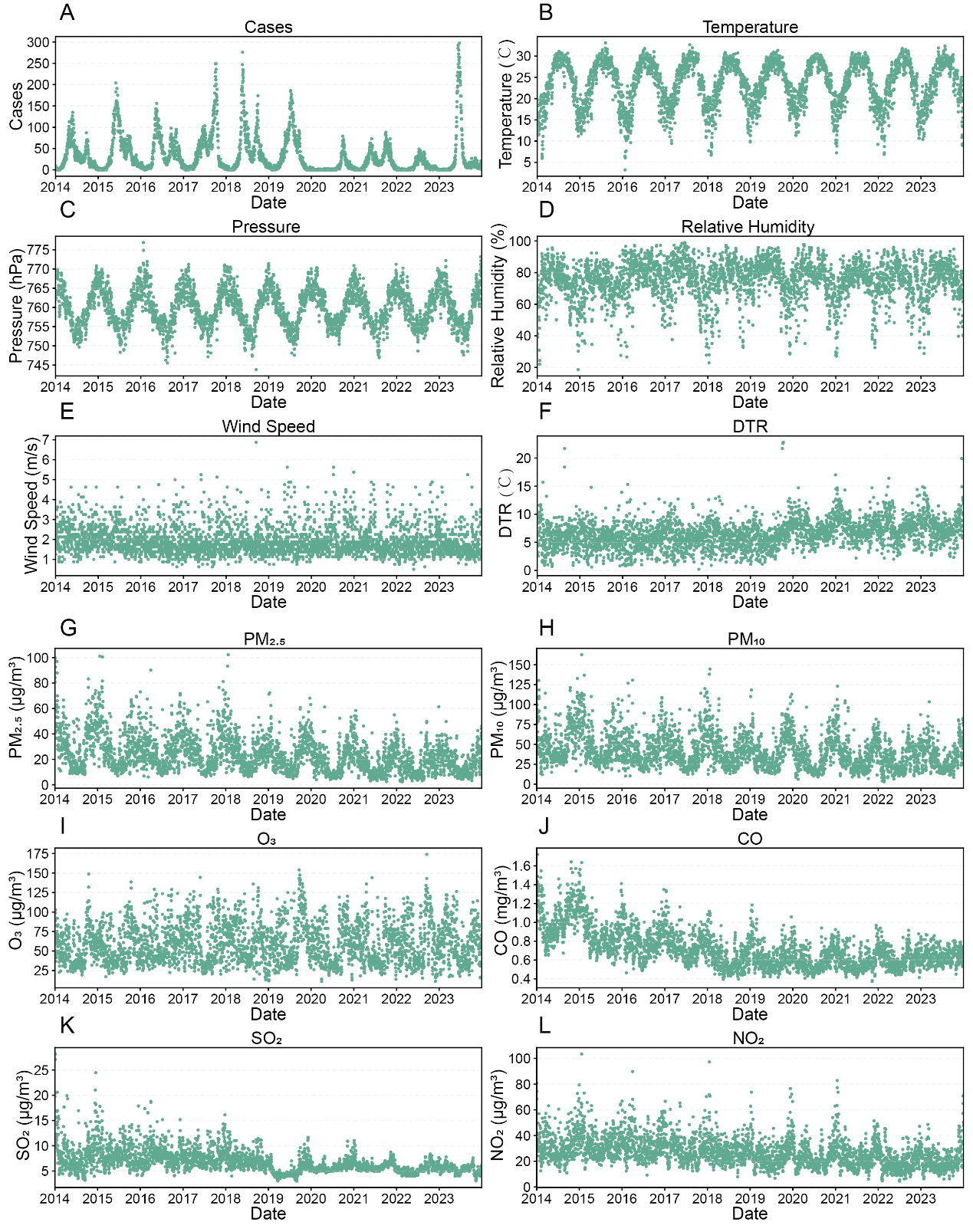


**Figure S1.** The temporal distribution of daily HFMD cases and environmental factors in Bao’an district of Shenzhen from 2014 to 2023. A) Number of daily cases of HFMD; B) Daily Average Temperature; C) Daily Average Air Pressure; D) Daily Average Relative Humidity; E) Daily Average Wind Speed; F) Daily Average Temperature Range; G) Daily Average PM₂.₅; H) Daily Average PM₁₀; I) Daily Average O₃; J) Daily Average CO; K) Daily Average SO₂; L) Daily Average NO₂
