# Supplementary material for: Hand, Foot, and Mouth Disease Risk Prediction in Southern China: Time Series Study Integrating Web-Based Search and Epidemiological Surveillance Data
Source: JMIR Infodemiology. 2025 Oct 9;5:e75434. doi: 10.2196/75434 (PMC12510436; doi:10.2196/75434)
Supplement: Multimedia Appendix 8 [file infodemiology-v5-e75434-s008.docx]

Multimedia Appendix 8


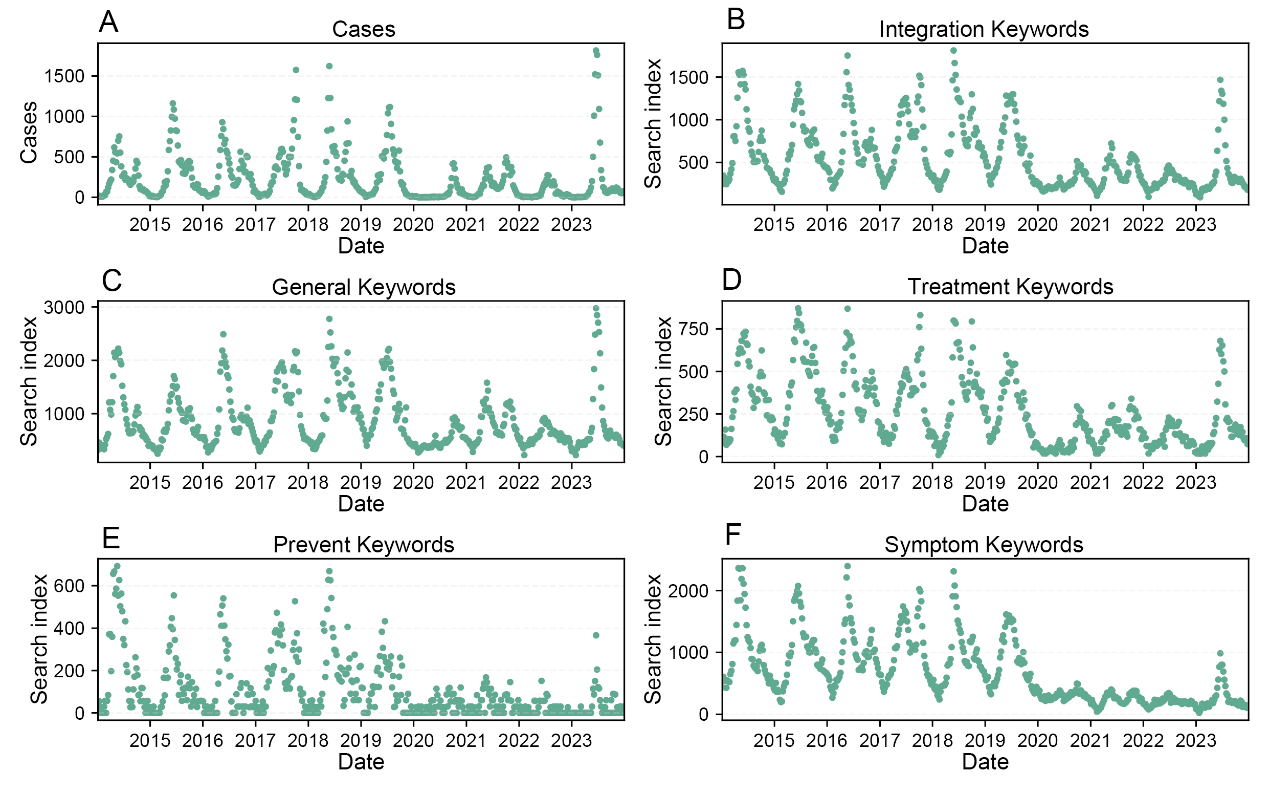


**Figure S1.** The temporal distribution of weekly HFMD cases and Baidu index in Bao’an District of Shenzhen from 2014 to 2023.
