# Supplementary material for: Hand, Foot, and Mouth Disease Risk Prediction in Southern China: Time Series Study Integrating Web-Based Search and Epidemiological Surveillance Data
Source: JMIR Infodemiology. 2025 Oct 9;5:e75434. doi: 10.2196/75434 (PMC12510436; doi:10.2196/75434)
Supplement: Multimedia Appendix 9 [file infodemiology-v5-e75434-s009.docx]

Multimedia Appendix 9

Table S1. Descriptive statistics of the HFMD cases and systematic factors from 2014 to 2023.

|  | Category | Factors | P25 | P50 | P75 | Mean | Standard deviation | Correlation coefficients |
| --- | --- | --- | --- | --- | --- | --- | --- | --- |
|  | HFMD | Cases | 3 | 16 | 46 | 32.53 | 43.11 | 1 |
|  | Meteorological factors | Temperature | 19.79 | 24.79 | 28.33 | 23.70 | 5.31 | 0.62 |
|  |  | Air pressure | 756.08 | 759.81 | 763.5 | 759.84 | 4.92 | -0.56 |
|  |  | RH ^a^ | 69.50 | 77.00 | 83.12 | 75.12 | 12.18 | 0.20 |
|  |  | Wind speed | 1.50 | 1.75 | 2.25 | 1.93 | 0.73 | 0.01 ^i^ |
|  |  | DTR ^b^ | 5.00 | 6.50 | 7.92 | 6.54 | 2.41 | -0.32 |
|  | Air pollutants | PM_2.5_ | 13.00 | 20.98 | 31.50 | 23.88 | 14.43 | -0.20 |
|  |  | PM_10_ | 25.54 | 36.92 | 53.16 | 41.35 | 21.34 | -0.19 |
|  |  | SO_2_ | 5.20 | 6.16 | 7.70 | 6.67 | 2.30 | 0.13 |
|  |  | NO_2_ | 19.00 | 25.04 | 32.58 | 26.86 | 11.13 | -0.05 |
|  |  | O_3_ | 37.78 | 55.22 | 76.15 | 59.20 | 25.64 | -0.09 |
|  |  | CO | 0.56 | 0.67 | 0.81 | 0.71 | 0.20 | -0.02 ^i^ |
|  | PHSMs ^c^ | PHSMs | 0 | 0 | 52.60 | 17.65 | 27.39 | -0.37 |
|  | Baidu Index | Composite index ^d^ | 273.73 | 427.39 | 735.15 | 553.09 | 365.27 | 0.87 |
|  |  | General ^e^ | 491.24 | 678.12 | 1103.87 | 877.28 | 540.32 | 0.89 |
|  |  | Symptoms ^f^ | 256.01 | 522.64 | 999.29 | 677.48 | 517.42 | 0.73 |
|  |  | Treatment ^g^ | 101.11 | 202.56 | 386.42 | 263.66 | 202.27 | 0.83 |
|  |  | Prevention ^h^ | 0 | 57.00 | 147.60 | 106.89 | 142.05 | 0.68 |

^a^ RH, relative humidity. ^b^ DTR, Daily temperature range. ^C^ PHSMs, public health and social measures. All indicators are on a daily scale, with the correlation coefficients calculated using Spearman’s rank correlation. The exceptions are those labeled with letters (^d-h^), which are on a weekly scale and have correlation coefficients calculated using Pearson’s correlation. The statistical test P-values for all correlation coefficients were less than 0.05, except for that with letter “i”.
