# Supplementary material for: Hand, Foot, and Mouth Disease Risk Prediction in Southern China: Time Series Study Integrating Web-Based Search and Epidemiological Surveillance Data
Source: JMIR Infodemiology. 2025 Oct 9;5:e75434. doi: 10.2196/75434 (PMC12510436; doi:10.2196/75434)
Supplement: Multimedia Appendix 11 [file infodemiology-v5-e75434-s011.docx]

Multimedia Appendix 11


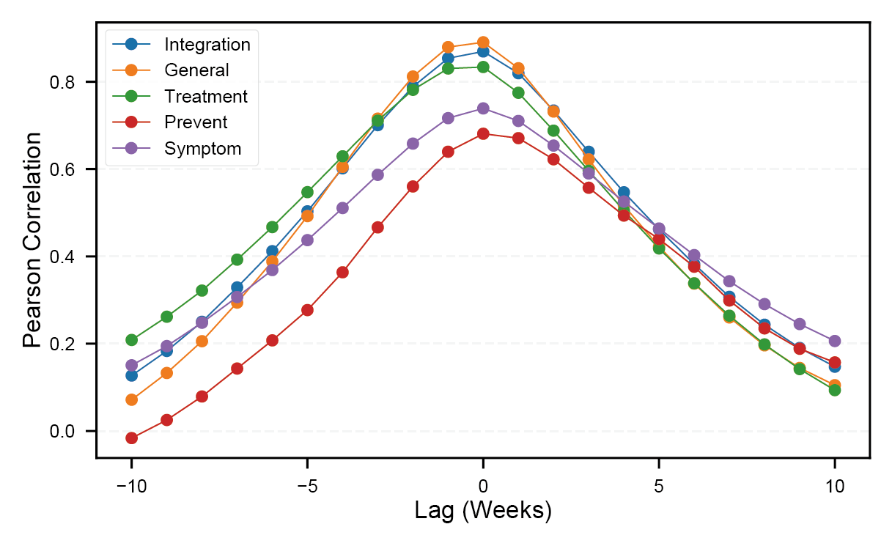


**Figure S1.** Cross-correlation coefficients between weekly number of HFMD cases and different groups of Baidu index composite Terms. Integration: A composite index for all HFMD-related terms; General: Baidu Index for General HFMD Terms; Treatment: A composite index for Treatment-Related Terms; Prevent: A composite index for Prevention-Related Terms; Symptom: A composite index for Symptom-Related Terms.
