# Supplementary material for: Hand, Foot, and Mouth Disease Risk Prediction in Southern China: Time Series Study Integrating Web-Based Search and Epidemiological Surveillance Data
Source: JMIR Infodemiology. 2025 Oct 9;5:e75434. doi: 10.2196/75434 (PMC12510436; doi:10.2196/75434)
Supplement: Multimedia Appendix 12 [file infodemiology-v5-e75434-s012.docx]

Multimedia Appendix 12


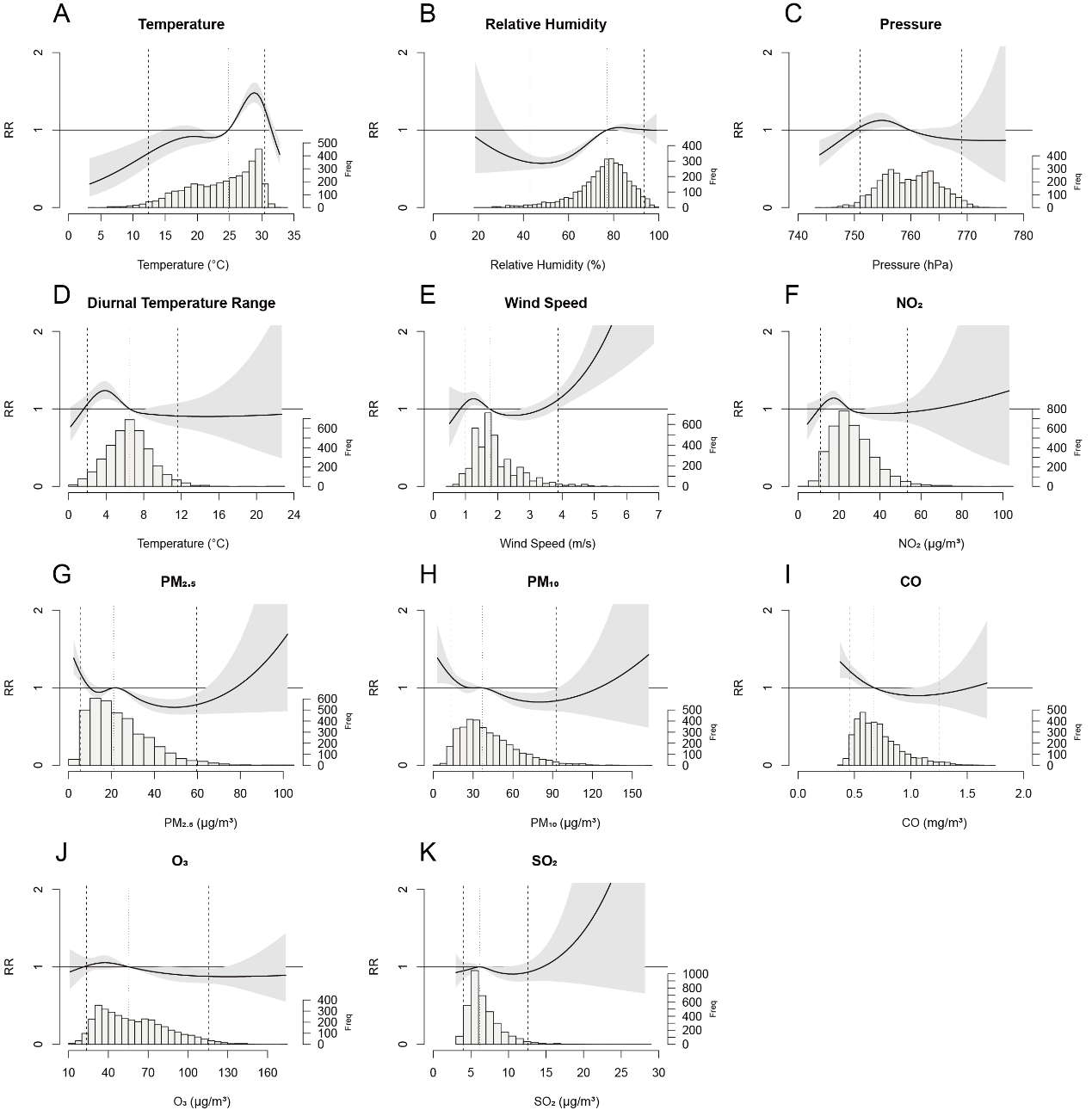


**Figure S1.** Cumulative lag effect plots of the impacts of environmental factors on the risk of HFMD.

A) Temperature; B) Relative humidity; C) Air pressure; D) Daily temperature range; E) Wind speed; F)NO_2_; G) PM_2.5_; H)PM_10_; I)CO; J)O_3_; K)SO_2_. The exposure–response associations are depicted as the best linear unbiased predictions (with 95% empirical confidence intervals, shaded in gray) of relative risk (RR) for Bao'an District, along with the distributions of related environmental factors. The dashed gray lines indicate the 2.5th, 50th, and 97.5th percentiles.
