# Supplementary material for: Hand, Foot, and Mouth Disease Risk Prediction in Southern China: Time Series Study Integrating Web-Based Search and Epidemiological Surveillance Data
Source: JMIR Infodemiology. 2025 Oct 9;5:e75434. doi: 10.2196/75434 (PMC12510436; doi:10.2196/75434)
Supplement: Multimedia Appendix 13 [file infodemiology-v5-e75434-s013.docx]

Multimedia Appendix 13

Table S1. Evaluation metrics for SARIMA model and advanced machine learning methods for 1-week forecasts, 2-week forecasts, 3-week forecasts, and 4-week forecasts for 2023 data.

| Time period | model | R^2 e^ | CORR ^f^ | MAE ^g^ | RMSE ^h^ |
| --- | --- | --- | --- | --- | --- |
| 1-week | SARIMA ^a^ | 0.95 | 0.98 | 53.34 | 99.31 |
|  | XGBoost ^b^ | 0.90 | 0.96 | 71.48 | 141.01 |
|  | LightGBM ^c^ | 0.90 | 0.96 | 65.09 | 141.61 |
|  | RF ^d^ | 0.72 | 0.88 | 117.60 | 246.45 |
|  | Stacking | 0.93 | 0.97 | 62.92 | 121.25 |
| 2-week | SARIMA | 0.83 | 0.92 | 91.75 | 190.59 |
|  | XGBoost | 0.80 | 0.91 | 100.47 | 203.81 |
|  | LightGBM | 0.78 | 0.90 | 103.97 | 214.96 |
|  | RF | 0.61 | 0.81 | 142.94 | 288.63 |
|  | Stacking | 0.83 | 0.92 | 87.84 | 185.08 |
| 3-week | SARIMA | 0.65 | 0.83 | 124.38 | 273.20 |
|  | XGBoost | 0.68 | 0.84 | 124.75 | 258.95 |
|  | LightGBM | 0.71 | 0.85 | 118.10 | 249.00 |
|  | RF | 0.48 | 0.71 | 167.12 | 334.17 |
|  | Stacking | 0.75 | 0.87 | 112.41 | 229.13 |
| 4-week | SARIMA | 0.31 | 0.69 | 169.79 | 383.69 |
|  | XGBoost | 0.58 | 0.77 | 142.13 | 298.75 |
|  | LightGBM | 0.61 | 0.78 | 140.40 | 288.52 |
|  | RF | 0.36 | 0.61 | 190.24 | 367.89 |
|  | Stacking | 0.64 | 0.80 | 132.47 | 276.81 |

^a^SARIMA, seasonal autoregressive integrated moving average. ^b^ XGBoost, extreme gradient boosting. ^c^ LGBM, Light Gradient Boosting Machine. ^d^ RF: random forest. ^e^ R^2^, coefficient of determination. ^f^ CORR, pearson correlation coefficient. ^g^ MAE, mean absolute error. ^h^ RMSE, root mean squared error.
