# Supplementary material for: Hand, Foot, and Mouth Disease Risk Prediction in Southern China: Time Series Study Integrating Web-Based Search and Epidemiological Surveillance Data
Source: JMIR Infodemiology. 2025 Oct 9;5:e75434. doi: 10.2196/75434 (PMC12510436; doi:10.2196/75434)
Supplement: Multimedia Appendix 14 [file infodemiology-v5-e75434-s014.docx]

Multimedia Appendix 14

Table S1. Accuracy evaluation of risk assessment for 1- to 4-week-ahead forecasts in 2023.

| Time scale of forecasts | Correct predictions (n/N) | Acuuracy | Overestimation (n/N) | Overestimation  rate | Underestimation(n/N) | Underestimation  rate |
| --- | --- | --- | --- | --- | --- | --- |
| 1-week | 50/52 | 96 | 2/52 | 4 | 0/52 | 0 |
| 2-week | 45/52 | 87 | 4/52 | 8 | 3/52 | 6 |
| 3-week | 46/52 | 88 | 4/52 | 8 | 2/52 | 4 |
| 4-week | 43/52 | 83 | 6/52 | 11 | 3/52 | 6 |
